# A Novel Viable Allele of Arabidopsis *CULLIN1* Identified in a Screen for *Superroot2* Suppressors by Next Generation Sequencing-Assisted Mapping

**DOI:** 10.1371/journal.pone.0100846

**Published:** 2014-06-23

**Authors:** Daniel I. Pacurar, Monica L. Pacurar, Andrea M. Pacurar, Laurent Gutierrez, Catherine Bellini

**Affiliations:** 1 Department of Plant Physiology, Umeå Plant Science Centre, Umeå University, Umeå, Sweden; 2 Faculty of Horticulture, University of Agricultural Sciences and Veterinary Medicine, Cluj Napoca, Romania; 3 Molecular biology platform (CRRBM), Université de Picardie Jules Verne, Amiens, France; 4 Institut Jean-Pierre Bourgin, French National Institute for Agricultural Research (UMR1318 INRA-AgroParisTech), Versailles, France; 5 Present address: SweTree Technologies AB, Umeå, Sweden; Emory University School Of Medicine, United States of America

## Abstract

Map-based cloning (MBC) is the conventional approach for linking phenotypes to genotypes, and has been successfully used to identify causal mutations in diverse organisms. Next-generation sequencing (NGS) technologies offer unprecedented possibilities to sequence the entire genomes of organisms, thereby in principle enabling direct identification of causal mutations without mapping. However, although mapping-by-sequencing has proven to be a cost effective alternative to classical MBC in particular situations, methods based solely on NGS still have limitations and need to be refined. Aiming to identify the causal mutations in suppressors of *Arabidopsis thaliana superroot2* phenotype, generated by ethyl methane sulfonate (EMS) treatment, we combined NGS and classical mapping, to rapidly identify the point mutations and restrict the number of testable candidates by defining the chromosomal intervals containing the causal mutations, respectively. The NGS-assisted mapping approach we describe here facilitates unbiased identification of virtually any causal EMS-generated mutation by overlapping the identification (deep sequencing) and validation (mapping) steps. To exemplify the useful marriage of the two approaches we discuss the strategy used to identify a new viable recessive allele of the Arabidopsis *CULLIN1* gene in the non-reference Wassilewskija (Ws-4) accession.

## Introduction

Map-based cloning (MBC) has been, and still is, widely used to identify genetic changes underlying mutant phenotypes in diverse organisms. It is a powerful technique with well-proven robustness [1], although traditional mapping experiments are generally labor intensive and hampered by needs for inter-accession crosses and selection of recombinants in the following generation(s) for mapping [2]–[4]. However, mapping mutations in well-established model organisms like Arabidopsis is much facilitated by the availability of a substantial genetic toolbox including an entire annotated reference genome, sequenced alternative accessions, and a multitude of marker systems [5]. In addition, several methods for identifying ethyl methane sulfonate (EMS)-induced point mutations in Arabidopsis and various other organisms have been developed using whole genome (re) sequencing following advances and reductions in cost of next generation sequencing (NGS) technologies. This has accelerated the process of identifying causal mutations, but methods based solely on NGS data still have limitations and need to be refined.

The NGS-based methods can be divided into two main types. The first is usually referred to as mapping-by-sequencing. The included methods all combine bulk segregant analysis (pooling recombinant genomes) with whole-genome sequencing (WGS) [1], [6]–[10]. Their main advantage over classical MBC is that they allow simultaneous mapping and mutant identification, by analyzing NGS-generated data from a pool of recombinant F_2_ individuals and subtracting the putative causal mutations after comparing the sequences to a reference genome. Alternatively, bulked segregants in the same accession can be used for deep sequencing [11]–[14]. The second group of methods all use a more direct approach, direct sequencing of mutant genomes and subsequent identification of causative EMS-induced mutations by comparing them to a reference genome, thereby eliminating the need for outcrossing [15]. However, a reference genome is needed as a scaffold in both approaches, although a method based on comparing *k*-mers in WGS datasets that eliminates the need for segregating populations and reference sequences has been recently described [16].

The full potential of the abovementioned methods can only be exploited in particular situations (e.g. when two or more alleles are isolated in the same screen) and/or require specialized software. Here we used a reliable alternative that can be used when single or multiple alleles are identified in the same screen, in reference or non-reference accession backgrounds. Furthermore, by sequencing a pool of homozygous F_3_ mutants, with or without prior back-crossing, it eliminates the risk of misscoring mutant plants in a recombinant segregating population that might occur due to incomplete penetrance of the mutant phenotype. By combining the respective advantages of deep sequencing with mapping virtually any causal mutation induced by EMS can be identified and validated. NGS-assisted mapping uses the full advantages of NGS to identify point mutations in EMS-induced mutants, and of classical mapping to restrict the number of testable candidates and define the chromosomal interval containing each causal mutation. In most situations, this second step can be achieved by coarse mapping, which is now straightforward in Arabidopsis [5].

Using a classical MBC approach, assisted by *in silico* identification of new INDEL (INsertions/DELetions) markers, we isolated a number of genes in a non-reference *A. thaliana* accession [5], [17]. To identify the causal mutations in the remaining selected mutants, we combined the available course mapping information [17] with the WGS data obtained for the mutants, and identified the causal mutations in the first two mutants sequenced so far. In the example presented here we report the identification of a new recessive mutation in the Arabidopsis *CULLIN1* (*CUL1*) gene in the Wassilewskija (Ws-4) accession.

Protein turnover in plants, and other eukaryotes, is mediated via the ubiquitin/26S proteasome, which specifically regulates the degradation of key proteins in response to environmental and biological signals. Protein ubiquitination involves the coordinated action of three enzymes: ubiquitin-activating enzyme (E1), ubiquitin-conjugating enzyme (E2), and ubiquitin-ligating enzyme (E3), [18]. The CULLIN-RING E3 ubiquitin ligases (CRLs) control multiple aspects of plant development and adaptation, including hormone and light perception, regulation of the cell cycle and response to biotic and abiotic stimuli [19]. Plants synthesize three main types of CULLIN (designated CUL1/CUL2a/b, CUL3a/b, and CUL4 in Arabidopsis) [20], [21], each of which assembles distinct CRL complexes. Arabidopsis CULLIN 1 (CUL1) is part of the SCF, a four-subunit complex, where it acts as a scaffold for SKP1 (ASK) and RING-box 1 (RBX1). The substrate specificity of the complex is given by an F-box protein, which binds to the complex through its association with ASK [18].

In Arabidopsis, initially only embryo lethal *CUL1* semi-dominant null mutations were identified [22], greatly hindering efforts to probe roles of the SCFs (Skp1-CUL1-F-box protein containing complexes) and CUL1 in later developmental phases. A significant advance in CUL1 characterization was enabled by isolation of viable, recessive, weak alleles. Three of these alleles (*axr6-3* [23], *cul1-6* [18] and *cul1-7* [24]) are in Columbia-0 (Col-0) background and one, *icu13*, was recently isolated in Enkheim-2 (En-2) background [25]. All these alleles share pleiotropic developmental defects, affecting both seedling and adult morphology, but they also have phenotypic differences that have not been reconciled, demonstrating the complexity of SCF functions in plants and strongly indicating that understanding the full extent of CUL1 functions in plant growth and development would benefit from the identification of multiple *CUL1* alleles [18], [24]. In a screen for suppressors of the *superroot2* mutation of *A. thaliana* we identified a new viable weak allele mutant of the *CUL1* gene, which we describe in this article.

## Materials and Methods

### Mutant Screen and Physical Mapping

The *superroot2* suppressor mutant designated *494* was identified in an EMS-mutagenized homozygous *sur2-1gl1* population [17]. Its adventitious rooting-related phenotypic characterization and coarse genetic mapping have been recently described [17]. Briefly, genetic mapping was accomplished using 160 phenotyped mutant plants collected from a F_2_ population derived from a cross between *494* (Wassilewskija 4, Ws-4 background) and *atr4-1*, an allele of *sur2* in Columbia-0 (Col-0) background [26]. The mapping strategy and the molecular markers used have been previously described [5].

### DNA Preparation and Sequencing

Approximately 1 g samples were collected from pools of 25 homozygous *sur2-1gl1*, homozygous *494* (F_3_ generation after the 4^th^ backcross) and homozygous *2035* (F_3_ generation after the 2^nd^ backcross) mutant plants, respectively, and bulked. DNA was extracted from each sample as described by Hanania et al. [27] and eluted in 200 µl of water. The concentration and quality of the DNA were determined using a NanoDrop 2000 spectrophotometer (Thermo Scientific) then the samples were freeze-dried for shipping. Extracts from each line containing more than 5 µg of high-quality DNA, meeting the quality requirements for sequencing, were sequenced at Beijing Genomics Institute Shenzhen (BGI) using the Illumina HiSeq 2000 high-throughput sequencing system. The sequencing conditions, data processing procedures and alignment results are summarized in Tables S1 and S2 in File S1. DNA libraries covering 4.29 giga base pairs (Gb), 4.54 Gb and 4.29 Gb (clean data) were generated for *sur2-1gl1*, *494* and *2035,* respectively. Very high quality data were obtained from all samples, with Q20 values (error rate <1%) of 96.88 for *sur2-1gl1*, 96.2 for *494* and 96.8 for *2035*, respectively. The deep-sequencing datasets have been submitted to the European Nucleotide Archive (ENA), and are available through the following link: https://www.ebi.ac.uk/ena (accession ID: ERA296179).

### Analysis of the Sequence Data

The Col-0 (TAIR10) sequence was used as reference genome, while both *sur2-1gl1* and *494* are in Wassilewskija-4 (Ws-4, N5390) background. Consequently, to construct a Ws-4 reference genome for the mutant *494*, sequencing reads of the parental genotype *sur2-1gl1* were first aligned against the reference genome sequence (TAIR10) using SOAP2 (http://soap.genomics.org.cn/soapaligner.html), and SNPs of the sequenced genome were detected using SOAPsnp (http://soap.genomics.org.cn/soapsnp.html). Using SNPs from the *sur2-1gl1* sequence the corresponding sites in the TAIR10 were replaced, and the newly constructed *sur2-1gl1* genome was subsequently used as reference for *494*. To identify the *494*-specific mutation, credible SNPs (which are likely mutations between *494* and *sur2-1gl1*) were filtered from credible loci differing between *494* and *sur2-1gl1*-TAIR10. These are relatively reliable loci filtered using the following criteria: consensus quality ⩾20 (error rate <1%), total depth ⩾5 and ⩽50 (to avoid copy number variation, CNV), and estimate copy number of the site <2. Only homozygous *494* SNPs were considered further. We used the same approach to identify the causal mutation in the mutant *2035* (data not shown).

### Confirmation of the Causal Point Mutation in *494* and Genotyping

The putatively causal mutation identified in the mapped region of *494* by analyzing the NGS dataset was subsequently re-confirmed by Sanger sequencing. To confirm the splicing defect and intron retention in the *494* mRNA, total RNA was extracted from seedlings of both *494* and *sur2-1gl1* mutants using the RNAquous isolation kit (Ambion), treated with rDNase I, using the DNA-*free* Kit (Ambion), and subsequently cDNA was synthesized using the iScript cDNA Synthesis Kit (BIO-RAD), according to the manufacturers instructions. Furthermore, gene-specific primers (At4g02570.F_TGGCTATCCCGCTTCTTCTA and At4g02570.R_TTGCAAACACAACCAGCAAT) spanning the splicing site altered by the *494* mutation were used to amplify the cDNAs, using standard PCR procedures.

To genotype the *494* point mutation, new derived cleaved-amplified polymorphic sequence (dCAPS) primers (494+EcoNI.F_CTTGCCCTGATTACCTGTTGAA and 494+EcoNI.R_ GCCACTCTCTCCCTCTCCTT) were designed using dCAPS Finder 2.0 software (http://helix.wustl.edu/dcaps/dcaps.html) [28]. One mismatch (underlined) was introduced in the F primer to incorporate a restriction site in the PCR product of one allele. After amplification, the PCR products were digested with EcoNI (Fermentas Fast Digest) following the manufacturer’s recommendations and electrophoretically separated on a 4% agarose gel. The wild type yielded two fragments of 133 and 16 bp, respectively, while the *494* allele gave one band of 149 bp.

To genotype *sur2-1*, which carries a 61 bp insertion in the cytochrome P450 CYP83B1 gene [29], primers were designed (Sur2*-*1F_AGCTTGGTTTCGGACAGTACAC and Sur2*-*1R_ACTTAGATCAACGGTGCCTGAT) that amplify a 237 bp fragment in *sur2-1*, and a 176 bp fragment in wild type, respectively.

To genotype the *axr6-3* and *cul1-7* alleles used for complementation tests, we used the genotyping primers and conditions described by Gilkerson et al. [24].

### Phenotypic Evaluation

Mutants and corresponding wild-type plants cultivated *in vitro* were characterized and their auxin contents were quantified as previously described [17]. For phenotypic evaluation of soil-grown plants, seeds were first germinated *in vitro* then the resulting seedlings were transferred into pots, which were placed in growth chambers providing short day (8 h darkness/16 h light) conditions at 22°C/18°C (light/dark temperatures). Plants under all growth conditions were visually inspected at time-points corresponding to selected developmental stages.

## Results and Discussion

### Mutant Isolation and Mapping

Genetic and physiological studies have shown that adventitious root (AR) formation is a heritable quantitative genetic trait controlled by multiple endogenous and environmental factors (reviewed in: [30]–[33]). Little is known about the molecular mechanisms controlling this developmental process, but we have recently started to unveil the complex regulatory mechanisms controlling AR formation. Using *Arabidopsis thaliana* as a model, we have shown that auxin and light signaling play essential roles in regulating AR formation on Arabidopsis hypocotyls [34]–[36]. We have also obtained preliminary indications that different regulatory pathways control lateral root and AR initiation in the hypocotyl, although both types of roots originate from pericycle cells [37], [38].

To substantiate these findings we screened seedlings obtained from EMS-mutagenized homozygous *superroot2-1glabra1* (*sur2-1gl1*) seeds, aiming to identify Arabidopsis mutants that produce a nearly normal main root system but have specific impairment in AR formation on etiolated hypocotyls [17]. The mutant designated *494* was identified during that screen. The mutation it carries suppresses the AR phenotype of the *superroot2-1* (*sur2-1*) mutant [39], and the suppressor mutant develops significantly fewer AR on the hypocotyl than *sur2-1gl1*, despite retaining similarly high endogenous IAA levels [17].

Homozygous *494* mutant plants selected from a homozygous M_3_ population were backcrossed four times with the *sur2-1gl1* parental genotype to remove EMS-induced SNPs not associated with the phenotype. In parallel, homozygous *494* M_3_ mutant plants were outcrossed with the *atr4-1* mutant, carrying an allele of the *sur2* mutant in Col-0 background [26], to generate a mapping population. Lastly, to identify potential alleles, allelic tests were conducted by crossing the mutant with other mutants isolated in the screen.

**Figure 1:**
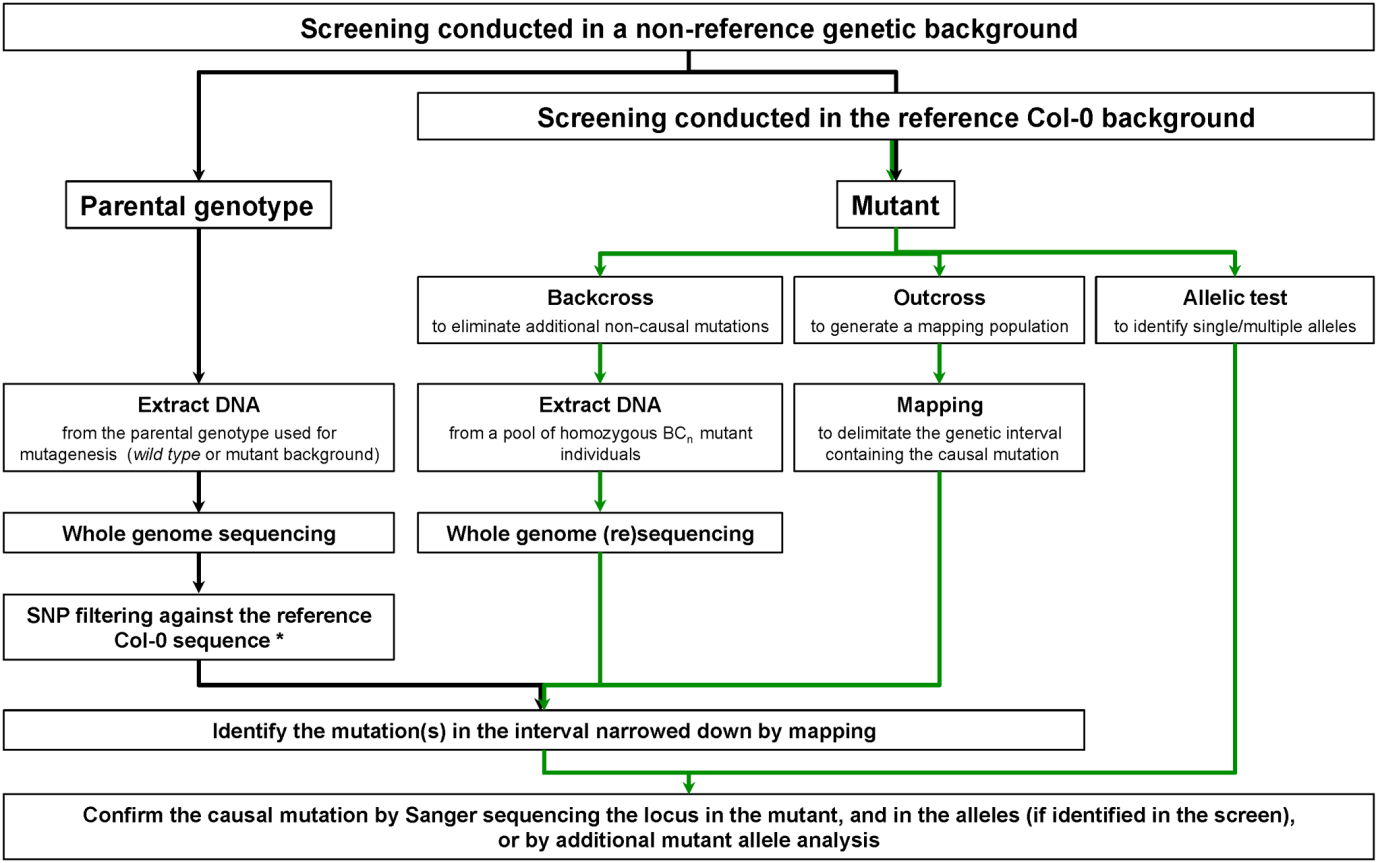
Identification of EMS-induced mutants by NGS-assisted mapping. For mutants identified in the reference Col-0 background, taking the green path leads to mutation identification. For mutants identified in a non-reference background, a parallel sequencing of the parental genotype (black path) is required. *Replacement of Col-0 specific SNPs with sites specific for the parental genotype, followed by use of the new constructed genome to extract the EMS-induced mutations in the mutant.

The *494* mutation was mapped on genomic DNA extracted from phenotyped mutant seedlings that produced fewer AR than *sur2-1gl1*, identified in a segregating F_2_ mapping population grown *in vitro* as previously described [5]. Coarse mapping was completed using newly identified INsertions/DELetions (INDEL) markers, as described in *Materials and Methods*. A flowchart of the NGS-assisted mapping approach, which can be applied for identifying mutations in both reference and non-reference Arabidopsis accession backgrounds, is shown in Figure 1.

### Identification of the Putative Causal Mutation in the Mapped Chromosomal Region

**Figure 2:**
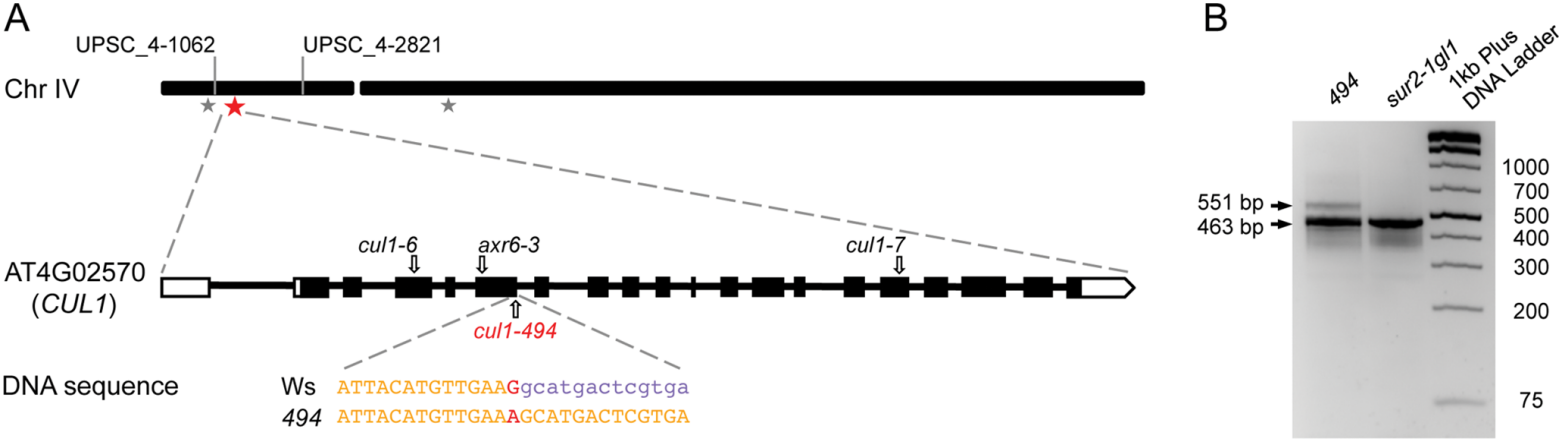
Annotation of putative causal mutations (A). Locations of EMS-induced mutations affecting the CDŚs on chromosome IV are marked with asterisks. The red asterisk indicate the mutation located in the region defined by mapping, which is flanked by the UPSC mapping markers [5]. Below, the structure of the *CUL1* gene is shown, indicating positions of known recessive mutations giving viable mutants used in this study for complementation tests. The position and nature of the *cul1-494* mutation, compared to wild type are highlighted on the DNA sequence. PCR amplification of the *494* and *sur2-1gl1* cDNAs with primers spanning the splicing site affected by the *cul1-494* mutation (B). A 463 bp fragment, corresponding to the correct spliced variant was detected in *sur2-1gl1*, while an additional 551 bp splicing variant was detected in *494*.

Even after applying the filtering regime described in *Materials and Methods* we identified 33 mutations scattered across the five chromosomes by comparing the *494* and newly constructed *sur2-1gl1* reference sequences, 25 of which were canonical C:G-to-T:A EMS-induced changes (Table S3 in File S1). Twenty-one of these mutations are situated in non-coding sequences (one in a 3′UTR, one in a 5′UTR, two in transposons, four in introns, and 13 in intergenic regions). Of the 12 mutations affecting the CDS, only six were non-synonymous. Interestingly, the only mutation located in the genomic interval 1.062.516 bp - 2.821.733 bp defined by the mapping markers UPSC_4*-*1062 and UPSC_4*-*2821 in the top of chromosome 4, is a synonymous G-to-A substitution at position 1.130.414 bp (Figure 2A). That mutation, located at the junction between the 5^th^ exon and the 6^th^ intron of the *CUL1* gene (AT4G02570), affects the splicing efficiency of the 6^th^ intron, as confirmed by comparing the PCR amplification products of the *sur2-1gl1* and *494* cDNAs (Figure 2B). Gene-specific primers spanning the splicing site amplified a 463 bp fragment in *sur2-1gl1*, while in addition to the correct spliced variant a larger 551 bp amplicon was detected in *494* (Figure 2B). The size difference of 88 bp between the *494* amplicons corresponds to the size of the 6^th^ intron. These observations show that although correct splicing occurs in *494* and some wild-type protein is produced, (probably less efficiently than in *sur2-1gl1*), the *494* point mutation leads to intron retention in the *494* mRNA, translation of which is predicted to yield a truncated protein product.

EMS mutagenesis typically induces hundreds of randomly distributed mutations per genome [3], [15] that can hamper the direct identification of causal mutations when using NGS data alone. Performing sufficient allelic tests (using existing alleles or T-DNA insertion lines), or complementing all of the candidates by transforming mutants with corresponding wild-type sequences would clearly be extremely tedious and generally unfeasible. The number of candidate mutations can be reduced by repeated backcrossing to the parental genotype before sequencing or by directly sequencing genomes carrying two or more independent alleles isolated in the same screen, followed by a search of unique mutations located in the same gene in multiple genomes. The first (repeated backcrossing) option is time-consuming, even if the focal organism has short generation times like Arabidopsis, and not always very efficient for removing EMS-induced SNPs that are not associated with the phenotype of interest. However, backcrossing is not essential for identifying EMS-induced mutations through NGS-assisted mapping, nor is the number of backcrosses important, since the strategy yielded very similar results when applied to mutants backcrossed four or two times (Table S3 and Table S4 in File S1; [40]). In the example discussed here, a high number of potential candidate mutations were identified after filtering, even if mutant plants were backcrossed four times before sequencing. Second, a typical non-saturating EMS mutagenesis screen provides the likelihood of detecting only single alleles [41], therefore direct sequencing of the mutant will have to be supported by mapping [12], [42]. Furthermore, in our particular case we found that mapping-by-sequencing is less suitable for identifying mutations with a weak phenotypic penetrance because it is difficult or even sometime impossible to trace mutant seedlings correctly in pooled F_2_ recombinants, or contaminants that occasionally occur [5]. Thus, sequencing such pools could have generated misleading or truncated information as described by [40] and sequencing a pool of homozygous F_3_ mutants turned to be a more reliable alternative.

### A New Viable Recessive Allele of the Arabidopsis *CUL1* Gene Identified in a Screen for *Superroot2* (*Sur2*) Suppressors

Confirmation by Sanger sequencing of the point mutation identified in the *CUL1* gene (AT4G02570), the only mutation situated in the mapped region, prompted us to consider it as a strong candidate for the *494* suppressor phenotype and we named this new allele *cul1-494*.

**Figure 3:**
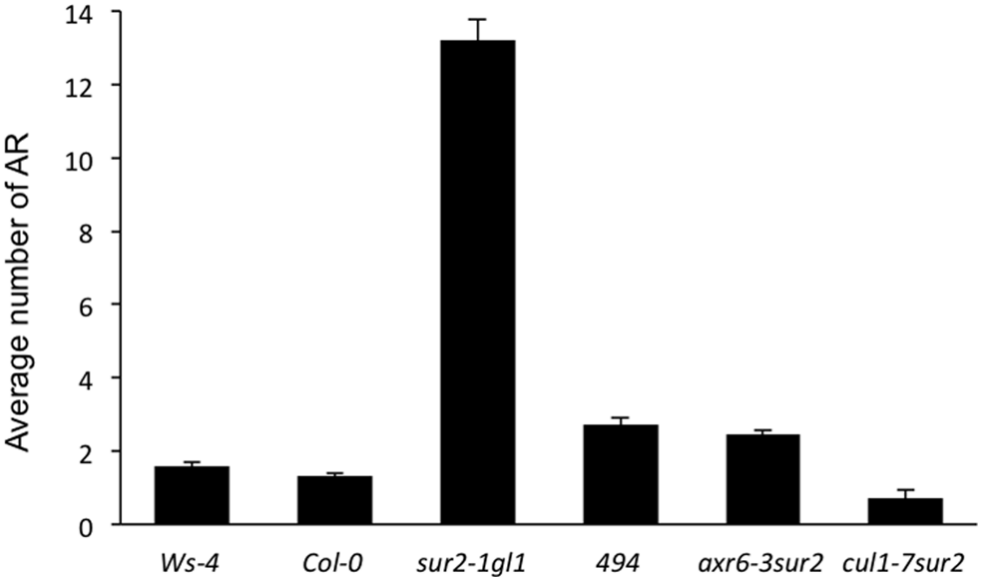
*axr6-3* and *cul1* *-* *7* mutations reduce the AR numbers produced by double mutants with *sur2*. The number of adventitious roots was counted on at least 35 seedlings of each line in two replicates and the data were pooled. Error bars indicate standard errors. One-way ANOVA and Tukey’s multiple-comparison post-tests indicate that the double mutants are not significantly different from their respective wild types (P<0.05; *n>*70).

To demonstrate unambiguously that the *cul1-494* mutation confers the suppressor phenotype we conducted complementation tests using three viable Col-0 alleles *axr6-3* [23], *cul1-6* [18], and *cul1-7* [24]. They all failed to complement *cul1-494*, confirming that *cul1-494* is a new viable allele of *CUL1*. Moreover, both *axr6-3sur2-1* and *cul1-7sur2-1* double mutants produce fewer AR than *sur2-1* (Figure 3), confirming that the *axr6-3* and *cul1-7* mutations suppress the AR phenotype of *sur2-1* in a similar way to *cul1-494*.

Phenotypically, *cul1-494* mutant differ in several respects from those carrying other known viable *CUL1* mutant alleles, notably its developmental defects are less pronounced. The first allele, *axr6-3* was isolated in a screen for *tir1-1* enhancers designed to identify genes required for SCF^TIR1^-mediated auxin responses [23]. *axr6-3* plants exhibit impaired auxin responses, reduced apical dominance, delayed senescence, reduced male fertility, and aberrant flower development. In addition, this allele is temperature-sensitive, mutant plants being sterile at 22°C, but fertile at 18°C. The *axr6-3* point mutation, located at the N terminus of the protein, interferes with Aux/IAA protein degradation and prevents the assembly of SCF^TIR1^ complexes by disrupting ASK1 binding. *cul1-6*, a recessive viable allele that affects interaction with the SCF regulatory protein CAND1 (CULLIN ASSOCIATED AND NEDDYLATION DISSOCIATED), was isolated in a screen for mutants resistant to sirtinol [18]. *cul1-6* plants have defects in seedling and adult morphology, including delayed leaf emergence, retarded root growth, reduced apical dominance, curled leaves and altered floral morphology. In addition to reduced auxin sensitivity, *cul1-6* seedlings display reduced sensitivity to other hormones including jasmonic acid, the cytokinin 6-benzyladenine and ethylene, and are hyposensitive to red and blue light [18]. The third, viable, missense, recessive Col-0 *CUL1* allele, *cul1-7*, was identified from a screen designed to isolate mutants with defective degradation of an Aux/IAA-luciferase (IAA1-LUC) fusion protein [24]. The *cul1-7* mutation affects subunit interaction at the CUL1 C-terminus. The mutant displays pleiotropic developmental defects similar to *axr6-3* and *cul1-6* (dwarfed with reduced apical dominance and curly leaves). Another viable *CUL1* allele with a point mutation located at the C-terminus, *icu13*, recently isolated in the Enkheim-2 (En-2) background, shows developmental defects that have been associated with alterations of auxin signaling [25]. Traits of the mutant plants include mild leaf hyponasty, increased numbers of vegetative leaves, early bolting (with short flower stems), and reduced apical dominance relative to wild-type.

**Figure 4:**
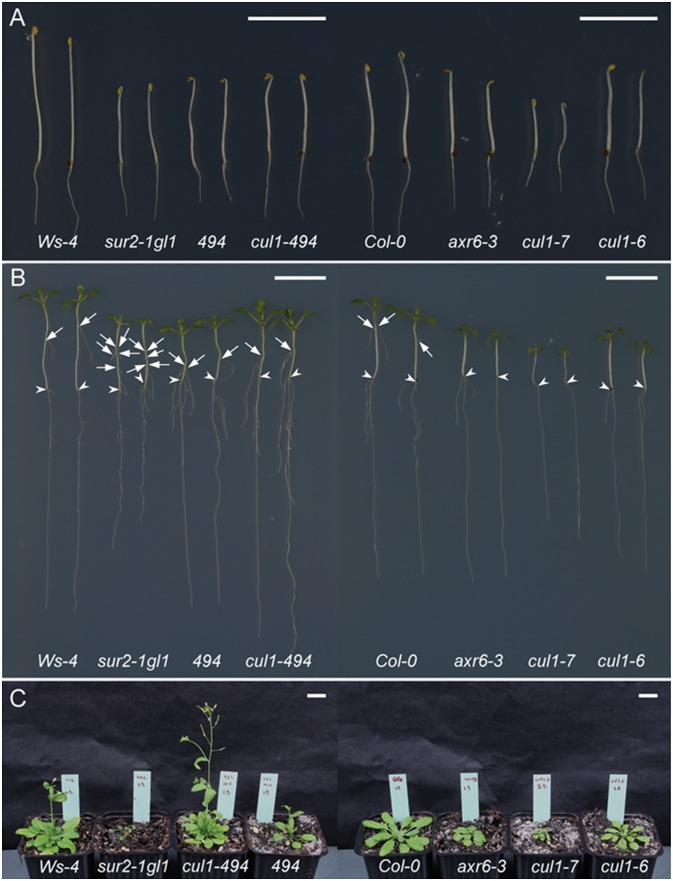
Seedling and rosette phenotypes of the *494* and *cul1* *-* *494* mutants. Seedlings were first etiolated in the dark for four days (**A**) and then transferred to the light for seven days (**B**). For phenotypic evaluation of soil-grown plants, seeds were first germinated *in vitro* and the resulting seedlings were subsequently transferred into pots, which were placed in growth chambers providing short day (8 h darkness/16 h light) conditions, at 22°C/18°C (light/dark temperatures) (**C**). Arrowheads indicate the root–hypocotyl junction; arrows indicate adventitious roots. Bars, 1 cm.

The suppressor mutant *494* was identified based on its AR-related phenotype, and characterized in connection to this developmental process [17]. A comparison of four-day old *in vitro* etiolated seedlings of *494* and *cul1-494* mutants with *axr6-3*, *cul1-7*, *cul1-6* and the parental controls Ws-4, *sur2-1gl1* and Col-0, respectively, is shown in Figure 4A. Both *494* [17] and *cul1-494* mutants displayed a reduced apical hook, like *axr6-3* and *cul1-6*. However, seven days after transfer to light both *494* and *cul1-494* grew better *in vitro* than *axr6-3*, *cul1-7* and *cul1-6* (Figure 4B). When grown in soil *cul1-494* flowers earlier than its control Ws-4 (Figure 4C), and like *axr6-3* [23] has reduced male fertility. In contrast, *494* is fertile, and except for a reduced number of rosette leaves and slightly reduced height, does not display any other obvious developmental defects, despite retaining similar endogenous auxin contents to *sur2-1gl1* [17]. These finding show that the *cul1-494* mutation not only suppresses the AR formation but also other developmental defects associated with high auxin content, such as epinastic cotyledons and leaves, long petioles and small leaf blades (Figure 4B and C). The *cul1-494* single mutant produces similar numbers of AR and lateral roots to the corresponding wild type, Ws-4, while *axr6-3*, *cul1-7*, *cul1-6* do not develop AR and produce fewer lateral roots than the wild type (Figure 4B). These observations suggest that *cul1-494* is a weaker allele than the others. Nevertheless, the suppression of AR production in *494* is likely due to weak auxin responses (since AtCUL1 is a component of SCF-type ubiquitin ligase complexes containing the F-box TIR1, which are essential for auxin responses [43]) arising from functional perturbation of SCF, as observed in the other mutants [18], [23]–[25]. This hypothesis is consistent with our previous findings that expression of three auxin-inducible *GH3* genes (*GH3.3*, *GH3.5*, and *GH3.6*) is strongly down-regulated in *494* suppressor mutant, despite its elevated endogenous auxin content [17]. These three *GH3* genes are essential for AR development in Arabidopsis hypocotyls [35], [36] and their expression level correlates with the number of ARs [17].

The *cul1-494* mutant shares, to a certain extent, the pleiotropic perturbations associated with the other viable *CUL1* mutant alleles and, more specifically, the fertility defect. AtCUL1 is a component not only of SCF-type ubiquitin ligase complexes containing the F-box TIR1 essential for auxin responses [43], but also complexes containing COI1, an F-box protein that mediates jasmonate signaling [44]. Furthermore, analyses of effects of point mutations in *AtCUL1* have shown it plays an essential role in responses to jasmonates [18], [45], which are important regulators of plant development and responses to environmental stresses [46]. Jasmonic acid (JA) has demonstrated importance for flower development and fertility [47], [48], and we recently showed that it inhibits AR formation downstream of auxin signaling in Arabidopsis hypocotyls [36]. Therefore, the low number of ARs produced by the suppressor mutant *494* is most likely due to a defect in SCF^TIR1^ functions, but not in SCF^COI1^ functions.

Our recent publication indicates that, in addition to auxin responses, ethylene biosynthesis is potentially impaired in the *494* mutant [17]. Interestingly, *cul1-6* also reportedly has reduced sensitivity to ethylene [18], and disturbed responses to several other hormones known to require SCF function have been observed in different *CUL1* mutants [18], [24]. This is consistent with observations that all the hormones known to be involved in the control of AR formation interact through complex crosstalk [33], which remains to be fully elucidated.

## Conclusions

To our knowledge, only two reports describe the identification of either a spontaneous [49] or EMS induced point mutation [15] in non-reference Arabidopsis accessions by NGS, and none promote the advantages of combining MBC with NGS to identify point mutations. Liu et al. [50] have used targeted parallel sequencing of defined genomic regions to identify Arabidopsis mutants, but although their method combines MBC with deep sequencing we found more advantageous to combine NGS and mapping. Our strategy has potentially the broadest applicability in Arabidopsis analyses for several reasons. Firstly, coarse mapping to the Arabidopsis genome is now straightforward, and facilitated by recent updates to TAIR (The Arabidopsis Information Resource; http://www.arabidopsis.org/) marker database [5]. Secondly, NGS and bioinformatics (which do not require extensive prior training to interpret), can be acquired at competitive costs by commercial service providers. Thirdly, combining the output of the two parallel complementary strategies can straightforwardly identify causal mutations.

The cited literature shows the unprecedented advantages of using NGS technologies to identify point mutations in various genetic backgrounds and model systems (e.g. [6], [42]). The multitude of methods developed by various research groups [1], [7]–[16] shows the versatility of NGS data sets, and the advantage of using them in combination with either established or new developed algorithms for mutation identification. Several recent articles describe the use of NGS data, exclusively, to identify causal mutations in EMS-induced mutants and eliminate reliance on labor-intensive classical mapping [1], [12]. However, although they provide examples of success, they also acknowledge the limitations of using NGS alone. Here we have shown that even in the NGS era classical mapping still provides valuable, complementary and robust data. The published strategies that completely exclude mapping may not be suitable in particular situations like the one we faced with low penetrant phenotype and only one allele identified. Arabidopsis mapping experiments are easily set up, and turned to be a valid option for defining genetic intervals containing causal mutations. The NGS-assisted mapping approach we describe here combines known techniques and provides a highly reliable alternative when only one allele is available [42].

The *cul1-494* allele we identified using NGS-assisted mapping is a valuable addition to the collection of weak-allele *CUL1* mutants, which will likely contribute to dissections of the role of CUL1 protein in plant development. It may be particularly helpful for studying the hormonal and ethylene signaling cross talk involved in AR formation in Arabidopsis.

## Supporting Information

File S1 Contains Table S1, Summary of the sequencing data production (Clean_Data). Table S2, Summary of the alignment results. Table S3, Annotation of homozygous mutations identified by sequencing the genome of *494* after four backcrosses. In bold, the causal suppressor mutation. Table S4, Annotation of homozygous mutations identified by sequencing the genome of *2035* after two backcrosses.
(DOCX)
