# Supplementary material for: A Novel Viable Allele of Arabidopsis CULLIN1 Identified in a Screen for Superroot2 Suppressors by Next Generation Sequencing-Assisted Mapping
Source: PLoS One. 2014 Jun 23;9(6):e100846. doi: 10.1371/journal.pone.0100846 (PMC4067405; doi:10.1371/journal.pone.0100846)
Supplement: File S1 — Contains Table S1, Summary of the sequencing data production (Clean_Data). Table S2, Summary of the alignment results. Table S3, Annotation of homozygous mutations identified by sequencing the genome of 494 after four backcrosses. In bold, the causal suppressor mutation. Table S4, Annotation of homozygous mutations identified by sequencing the genome of 2035 after two backcrosses. (DOCX) [file pone.0100846.s001.docx]

**Table S1** Summary of the sequencing data production (Clean_Data).

| **Genotype** | **Quality value >=20** | **Production** | |
| --- | --- | --- | --- |
|  | **Rate (%)** | **Reads (M)** | **Bases (Gb)** |
| *sur2-1gl1* | 96.88 | 47.72 | 4.29 |
| *494* | 96.20 | 50.49 | 4.54 |
| *2035* | 96.80 | 47.65 | 4.29 |

Note: “Clean data”: the data which have deleted adapter and removed the reads that the rate of low quality (quality value <=5) is more than or equal to 50% of these reads. M, million reads; Gb, giga base pairs.

**Table S2** Summary of the alignment results.

| **Genotype** | **Insert size**  **(bp)** | **Coverage rate**  **(%)** | **Mapped**  **data** | | **UniqMapped data** | | **Average depth** | |
| --- | --- | --- | --- | --- | --- | --- | --- | --- |
|  |  |  | **Reads rate**  **(%)** | **Base rate (%)** | **Reads rate**  **(%)** | **Base rate (%)** | **Sequencing (X)** | **Effective**  **(X)** |
| *sur2-1gl1* | 460 | 93.50 | 91.39 | 91.30 | 63.84 | 63.76 | 35.89 | 32.81 |
| *494* | 475 | 94.34 | 89.19 | 89.09 | 70.00 | 69.91 | 37.97 | 33.88 |
| *2035* | 471 | 94.05 | 90.29 | 90.18 | 71.40 | 71.30 | 35.83 | 32.36 |

Note: The genome size of TAIR10 is 119.67 Mb while the effective size is 119.48 Mb (not including the N base in the reference). Average sequencing depth = Clean data bases / the reference genome size. Average effective depth = Mapped data bases / the effective genome size.

**Table S3** Annotation of homozygous mutations identified by sequencing the genome of *494* after four backcrosses. In bold, the causal suppressor mutation.

| **Ch** | **Position** | **Mutation** | **Sequencing depth** | **Feature annotation** | **Mutated**  **codon** | **Mutated**  **amino acid** | **Type** | **Gene id** |
| --- | --- | --- | --- | --- | --- | --- | --- | --- |
| 1 | 100887 | G-to-A | 25 | CDS | GGA-to-AGA | G-to-R | Nonsyn | AT1G01240 |
|  | 114810 | G-to-A | 19 | CDS | GTG-to-GTA | V-to-V | Syn | AT1G01290 |
|  | 13627084 | C-to-G | 9 | Transposon | - | - | - | AT1G36260 |
| 2 | 9498172 | T-to-C | 18 | CDS | ATA-to-ACA | I-to-T | Nonsyn | AT2G22360 |
|  | 15555113 | T-to-C | 25 | 3`UTR | - | - | - | AT2G37035 |
| 3 | 222161 | C-to-T | 32 | CDS | AAG-to-AAA | K-to-K | Syn | AT3G01570 |
|  | 682611 | C-to-T | 22 | CDS | GAC-to-GAT | D-to-D | Syn | AT3G03030 |
|  | 2307400 | G-to-A | 29 | CDS | ATC-to-ATT | I-to-I | Syn | AT3G07250 |
|  | 2528826 | A-to-G | 30 | Intergenic | - | - | - | - |
|  | 3007848 | G-to-A | 34 | Intronic | - | - | - | AT3G09800 |
|  | 3375369 | G-to-A | 28 | CDS | CGG-to-CAG | R-to-Q | Nonsyn | AT3G10780 |
|  | 4300078 | G-to-A | 28 | CDS | GCG-to-ACG | A-to-T | Nonsyn | AT3G13290 |
|  | 5992709 | G-to-A | 14 | 5`UTR | - | - | - | AT3G17510 |
|  | 9337434 | C-to-A | 18 | Intergenic | - | - | - | - |
|  | 9840905 | G-to-A | 24 | CDS | GCT-to-GTT | A-to-V | Nonsyn | AT3G26750 |
|  | 9979474 | G-to-A | 12 | Intronic | - | - | - | AT3G27050 |
| 4 | 628659 | G-to-A | 26 | Intergenic | - | - | - | - |
|  | 833997 | C-to-T | 18 | CDS | CTC-to-CTT | L-to-L | Syn | AT4G01925 |
|  | **1130414** | **G-to-A** | **23** | **CDS** | **AAG-to-AAA** | **K-to-K** | **Syn** | **AT4G02570** |
|  | 1635305 | G-to-A | 18 | Transposon | - | - | - | AT4G03690 |
|  | 3427119 | C-to-T | 22 | Intergenic | - | - | - | - |
|  | 4854122 | T-to-G | 22 | Intergenic | - | - | - | - |
|  | 5520544 | T-to-A | 15 | Intergenic | - | - | - | - |
|  | 5599216 | C-to-T | 29 | CDS | GGT-to-GAT | G-to-D | Nonsyn | AT4G08770 |
|  | 5712042 | C-to-T | 26 | Intergenic | - | - | - | - |
|  | 5718505 | C-to-A | 12 | Intergenic | - | - | - | - |
|  | 6055177 | C-to-T | 14 | Intergenic | - | - | - | - |
|  | 9186808 | C-to-A | 12 | Intergenic | - | - | - | - |
| 5 | 22935555 | C-to-T | 36 | Intronic | - | - | - | AT5G56660 |
|  | 24835327 | C-to-T | 22 | Intronic | - | - | - | AT5G61820 |
|  | 24906145 | C-to-T | 24 | Intergenic | - | - | - | - |
|  | 25357936 | C-to-T | 17 | Intergenic | - | - | - | - |
|  | 26295563 | C-to-T | 27 | Intergenic | - | - | - | - |

**Table S4** Annotation of homozygous mutations identified by sequencing the genome of *2035* after two backcrosses.

| **Ch** | **Position** | **Mutation** | **Sequencing depth** | **Feature annotation** | **Mutated**  **codon** | **Mutated**  **amino acid** | **Type** | **Gene id** |
| --- | --- | --- | --- | --- | --- | --- | --- | --- |
| 1 | 13627084 | C-to-G | 9 | CDS | GGA-to-AGA | G-to-R | Nonsyn | AT1G01240 |
|  | 15216659 | T-to-C | 13 | Transposon | - | - | - | AT1G40127 |
|  | 16056590 | C-to-A | 20 | Transposon | - | - | - | AT1G42697 |
|  | 24475466 | A-to-T | 28 | CDS | GAT-to-GTT | D-to-V | Nonsyn | AT1G65800 |
| 5 | 65186 | A-to-C | 28 | Intergenic | - | - | - | - |
|  | 5631842 | T-to-C | 29 | Transposon | - | - | - | AT5G17125 |
|  | 12431671 | T-to-A | 23 | Transposon | - | - | - | AT5G33125 |
|  | 15481247 | G-to-A | 24 | Intronic | - | - | - | AT5G38690 |
|  | 15874695 | G-to-A | 9 | Intergenic | - | - | - | - |
|  | 16035276 | G-to-A | 19 | CDS | GAA-to-AAA | E-to-K | Nonsyn | AT5G40060 |
|  | 16317006 | G-to-A | 25 | Intergenic | - | - | - | - |
|  | 17108752 | G-to-A | 28 | CDS | GGT-to-AGT | G-to-S | Nonsyn | AT5G42670 |
|  | 17238058 | G-to-A | 31 | CDS | GCT-to-GTT | A-to-V | Nonsyn | AT5G42970 |
|  | 17651948 | G-to-A | 26 | Intergenic | - | - | - | - |
|  | 18149319 | G-to-A | 31 | Intergenic | - | - | - | - |
|  | 19218560 | G-to-A | 32 | Intergenic | - | - | - | - |
|  | 19952813 | G-to-A | 20 | Intergenic | - | - | - | - |
|  | 20196832 | G-to-A | 32 | mRNA | AAC-to-AAT | N-to-N | Syn | AT5G49710 |
|  | 20896589 | G-to-A | 16 | CDS | GAT-to-AAT | D-to-N | Nonsyn | AT5G51450 |
|  | 21766071 | G-to-A | 33 | mRNA | CAC-to-CAT | H-to-H | Syn | AT5G53580 |
|  | 22282208 | G-to-A | 19 | Intronic | - | - | - | AT5G54850 |
|  | 22430399 | G-to-A | 15 | CDS | ACC-to-ATC | T-to-I | Nonsyn | AT5G55310 |
|  | 22641127 | G-to-A | 25 | CDS | CCG-to-TCG | P-to-S | Nonsyn | AT5G55910 |
|  | 23609681 | G-to-A | 37 | CDS | GAT-to-AAT | D-to-N | Nonsyn | AT5G58410 |
|  | 24186916 | G-to-A | 8 | Intergenic | - | - | - | - |
|  | 24478071 | G-to-A | 30 | Intergenic | - | - | - | - |
